# Snakebite envenomation and community responses in an Amazonian floodplain: Public health and ethnobiological perspectives

**DOI:** 10.1371/journal.pgph.0006310

**Published:** 2026-04-15

**Authors:** Brenna Celina de Carvalho Muniz, Camila Raiane Sousa de Jesus, David Soares, Eduarda Gomes da Silva, Roberta Ferreira Abecassis, Síria Ribeiro, Alfredo P. Santos-Jr

**Affiliations:** 1 Programa de Pós-Graduação em Biodiversidade e Biotecnologia da Rede BIONORTE, Universidade Federal do Oeste do Pará (UFOPA), Santarém, Pará, Brazil; 2 Laboratório de Ecologia e Comportamento Animal (LECAN), Universidade Federal do Oeste do Pará (UFOPA), Santarém, Pará, Brazil; PLOS: Public Library of Science, UNITED STATES OF AMERICA

## Abstract

Snakebite envenomation is a serious tropical public health problem, with a high incidence in the Amazon region. In floodplain areas, the seasonal flood pulse intensifies interactions between humans and snakes, making the riverine populations of the Lower Amazon particularly vulnerable. This study analyzed how the riverine population of a floodplain community in the Lower Amazon perceives, identifies, and manages snakebite accidents, investigating the influence of cultural practices on access to formal medical care. A mixed-methods (qualitative-quantitative), cross-sectional, and descriptive-exploratory approach was adopted, based on ethnobiology. Semi-structured interviews were conducted with 39 residents of the Salvação community (Alenquer, Pará), followed by content analysis and descriptive statistics. Most participants (84.6%) had a personal or family history of snakebites. Local classification systems (ethnotaxonomy) revealed taxonomic divergences, such as the use of the term “*surucucu*” to describe *Bothrops atrox*. Although 33.3% sought hospital care, traditional treatments were prevalent, including the use of dolphin fat (*banha de boto*), traditional antidotes (*contravenenos*), and the ingestion of kerosene. Reported sequelae, such as persistent pain, were frequently associated with natural cycles such as the phases of the moon. Knowledge transmission on the subject is predominantly oral and centralized within the family network (66.7%). Informational gaps were identified, especially in domestic prevention and the use of harmful substances in first aid. The management of snakebite accidents in the floodplain is guided by complex ethnobiological knowledge, where traditional and formal medicine coexist as adaptation strategies. The findings reinforce the need for intercultural public health policies that integrate local knowledge with biomedical practices, aiming to reduce the time spent seeking hospital treatment and mitigating the risks associated with inadequate management behaviors.

## Introduction

Snakebite envenoming is a public health problem that affects tropical and subtropical regions [1]. Most of the world’s population, 5.8 million people, is prone to being bitten by a venomous snake. Every day, 7,400 people are bitten by snakes, and between 81,000 and 138,000 die as a result. About 400,000 people who were bitten presented sequelae such as permanent physical or psychological disabilities, including blindness, amputation, and post-traumatic stress disorder [2]. Given this scenario, the World Health Organization began developing a global strategy to reduce by half the number of deaths and disabilities caused by snakebites by 2030 [3].

A recent global estimate of snakebites showed 69.4/100,000 inhabitants per year and a mortality rate of 0.33/100,000 inhabitants per year. Stratifying this analysis by continent, Asia, Africa, and South America presented the highest incidences of snakebite accidents [4]. Previously, according to Chippaux [5], the average annual incidence of these accidents in the Americas was about 57,500 snakebite accidents (6.2 per 100,000 inhabitants) and mortality close to 370 deaths (0.04 per 100,000 inhabitants), and although this overall incidence was lower than in Asia and Africa, specific regions of Latin America were exceptions, such as the Amazon in the northern region of Brazil.

Brazil is divided into five large regions, one of which is the North region, composed of the states of Amapá, Amazonas, Acre, Pará, Rondônia, Roraima, and Tocantins. It is the largest region of the country in territorial extension and houses most of the Amazon rainforest [6]. According to the Information System for Notifiable Diseases (SINAN), in 2023, 32,595 cases of snakebite accidents and 143 deaths resulting from this type of occurrence were reported in Brazil. The North region presented an incidence rate of 51.84/100,000 inhabitants, approximately 3.4 times higher than the national average (15.28 accidents/100,000 inhabitants) [7].

The state of Pará was one of those that recorded the most snakebite accidents, with a total of 5,234 reported cases and 17 deaths, becoming the second state in Brazil with the highest number of deaths from snakebite accidents [7]. The Lower Amazon region, which covers 15 municipalities in the state of Pará, is also an area of high incidence of snakebite accidents. Accidents caused by the genus Bothrops (jararacas) predominate in this region, frequently associated with climatic factors (rains), occupational factors (rural work), and the male population, with a higher occurrence between 20 and 59 years [8].

Amazonian floodplain communities are environments that provide frequent encounters between humans and snakes. One of the reasons for this is the annual flood pulse that shapes daily activities, mobility and creates unique patterns of exposure and vulnerability [9–11]. During high and low water seasons, residents, known as “ribeirinhos,” are frequently in close contact with snakes, whether in their daily work activities involving fishing, extractivism, and livestock or even in domestic activities [12–14]. In these traditional communities, a fundamental aspect involves the ways snakes are locally perceived, named, and classified through systems based on long-term interaction with the environment and shaped by cultural knowledge.

Given the linguistic and taxonomic diversity of the region, the identification of species in clinical screening is often based on the signs and symptoms of the victims rather than scientific nomenclature [15–17]. These perceptions are also prevalent in Africa [18] and South Asia [19,20]. This influences treatment-seeking behavior [21], delays or facilitates access to formal medical care [22], and affects human attitudes toward snakes, mainly regarding the predation of these animals and their use for zootherapeutic practices [23].

Despite ophidism being widely known and studied throughout the Amazon, research in the Lower Amazon still presents gaps regarding the study of riverine populations’ perceptions of envenoming. Although there are relevant works in the region that contribute to the inventory of local snakes [24] and the epidemiological understanding of accidents [8,25], investigations from an ethnobiological perspective, aiming to hear the riverine population regarding how they shape the integral management of the accident, are still scarce.

This study seeks to fill this gap by analyzing how local management of snakebites accidents is shaped by culturally rooted practices. Due to the scarcity of data on floodplain communities in the Lower Amazon, we adopted an exploratory mixed-methods approach. To provide further depth regarding this dynamic, pre-defined statistical hypothesis were not tested, focusing instead on the inductive understanding of social phenomena. Therefore, the objective of this study was to examine how the riverine population of a floodplain community perceives, identifies, and manages snakebites and how these culturally rooted practices influence access to formal medical care while shaping local strategies for prevention and treatment.

Specifically, we explored (i) local identification and naming of venomous snakes and their influences on care-seeking decisions; (ii) cultural beliefs and traditional therapeutic practices, including the use of animal-based remedies (zootherapy); and (iii) reported health consequences and their perceived relationships with local environmental cycles. By integrating sociocultural and ecological perspectives, this work seeks to support intercultural health initiatives that recognize local knowledge as a critical component of comprehensive responses to neglected tropical diseases.

## Materials and methods

### Ethics statement

The study was approved by the Research Ethics Committee of the Universidade Federal do Oeste do Pará (UFOPA) and registered in the Plataforma Brasil database (Opinion No. 6,605,674) [26]. All procedures were conducted in compliance with the ethical principles of the Declaration of Helsinki and Resolution No. 466/2012 of the Brazilian National Health Council, which regulates research involving human beings. All participants were informed about the objectives and procedures of the study and provided written free and informed consent (TCLE) before the start of the interviews, ensuring anonymity and the right to withdraw at any time.

### Inclusion in global research

Additional information regarding specific ethical, cultural, and scientific considerations for inclusion in Global Research is included in the S2 Checklist.

### Study design

This is a mixed-methods (qualitative-quantitative), descriptive-exploratory, and cross-sectional study, grounded in the ethnobiological perspective. The design followed the COREQ (Consolidated Criteria for Reporting Qualitative Research) guidelines [27] to ensure rigor and transparency at all stages (S1 Checklist). This mixed-methods (qualitative-quantitative) approach integrated traditional narratives and ethnobiological knowledge through qualitative exploration while using descriptive statistics to characterize the sample profile, species citation frequencies and management practices.

### Research team and establishment of rapport

The interviews were conducted by a team of five researchers ([BCCM, CRSJ, DS, EGS, RFA], one man and four women), with backgrounds in biological sciences and master’s degrees in environmental sciences and biodiversity and conservation, in addition to vast experience in field herpetological research in the Brazilian Amazon. There was no prior relationship between the researchers and the residents. Aware of the community’s reserved profile, the team prioritized the establishment of rapport through informal dialogues and local coexistence before the formal interviews.

This insertion process was essential to mitigate response bias and ensure participant comfort. By clearly communicating their academic backgrounds and the goal of documenting local knowledge for public health purposes, the team established a transparent dialogue. Throughout the study, the researchers maintained a reflexive stance, balancing academic expertise with a learner’s attitude, and employed active listening to faithfully record traditional knowledge and identify public health challenges related to snakebites.

### Setting and study area

**Fig 1:**
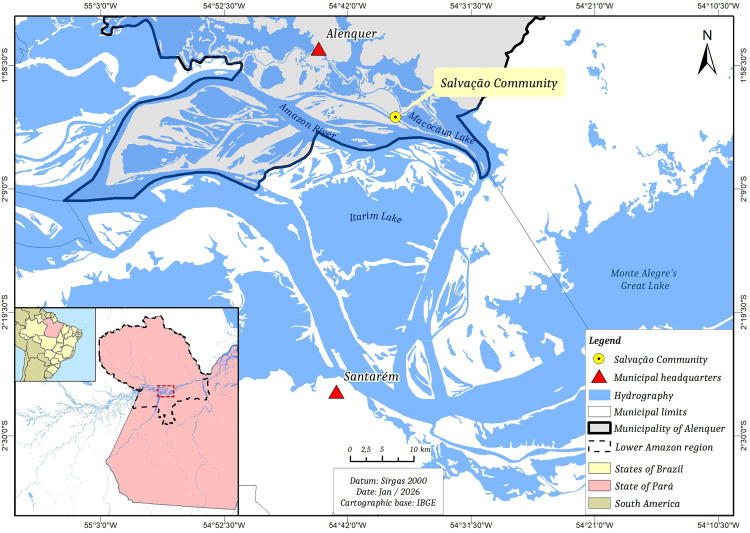
Location of the Salvação Community. Map showing the position of the Salvação floodplain community in the municipality of Alenquer, Pará State, in the Lower Amazon region of Brazil. Map produced by the authors using **QGIS 3.22 ‘Bialowiez’**. The administrative boundaries (base layers) were sourced from the **Instituto Brasileiro de Geografia e Estatística (IBGE)** [https://geoftp.ibge.gov.br/cartas_e_mapas/bases_cartograficas_continuas/bc250/versao2025/shapefile/bc250_shapefiles_2025_11_21.zip]. Terms of use available at: https://biblioteca.ibge.gov.br/visualizacao/livros/liv102169.pdf.

The study was conducted in the floodplain community of Salvação, located in the municipality of Alenquer, in the Santarém microregion, within the Lower Amazon region, state of Pará, Brazilian Amazon (2º02’53"S; 54º37’55"W) (Fig 1). The community comprises approximately 300 residents in 130 households, according to the local resident’s association. Livelihoods center on small-scale agriculture, artisanal fishing, and extensive livestock farming in floodplain areas.

Additional income is generated through small family businesses, including the sale of fuel, food, clothing, bread, jewelry, perfume, and drinking water [28]. The local ecology is defined by the annual flood pulse, with the peak flood between June and July and ebb periods from November to December, shaping mobility, subsistence activities, and exposure to wildlife [29].

The choice of the Salvação community was based on records from the Herpetological Collection of the Laboratório de Ecologia e Comportamento Animal (LECAN) at the Universidade Federal do Oeste do Pará (UFOPA). River travelers who dock in the Santarém microregion, Pará, frequently delivered dead specimens collected by floodplain residents, which served as a biological indicator of the interaction between snakes and humans in the region. Furthermore, in previous contact with local leaders, we found that the community had never received awareness actions regarding snakebite accidents, representing a health information void.

Logistics were also a decisive factor, as access to floodplain communities is difficult and expensive. Salvação, although remote, offered financial viability that allowed for the displacement of the four- member researcher team. The journey involved a 4-hour ferry trip from Santarém to Alenquer (69.7 km), followed by a 50-minute trip in a “rabeta” (a small, motorized boat typical of the region) until reaching the community.

### Participant selection and sampling

Intentional non-probabilistic sampling was adopted, operationalized through an active,exhaustive search in households. The established inclusion criteria were:

1. Representation of one resident per family unit.
2. Age over 18 years.
3. Fixed residence in the locality for at least 10 years, aiming to ensure the collection of perception and accumulated traditional knowledge.

The snowball sampling technique [30] was employed complementarily as an ethical strategy for community engagement in traditional populations, as being introduced by a member of the local community is often essential to establish trust and ensure the necessary openness for interviews. A total of 79 households were visited. Of these, 39 residents agreed to participate (representing 30% of the total dwellings in the community), a sample size determined by theoretical saturation and field logistical constraints. The remaining n = 40 households were not included due to either direct refusals or the absence of residents at the time of the visit. These instances were recorded in accordance with ethical precepts of respect and autonomy. Children and adolescents were excluded from the sampling process.

### Data collection procedure

Data collection took place between May 16 and 19, 2024 (S1 Data). Information was obtained through individual and face-to-face semi-structured interviews, in the Portuguese language, using a questionnaire (S1 Text). The choice of this technique was justified by the need to obtain in-depth data on local perceptions. Unlike closed-ended questionnaires, interviews allow participants to express their therapeutic itineraries in a narrative form, revealing essential cultural particularities [31,32]. The structure of the interviews was guided by a deductive approach, with thematic axes previously established from the ethnobiological and epidemiological literature (Table 1). However, the semi-structured nature of the script allowed for an inductive perspective during collection and analysis, enabling subthemes and peculiarities of the local reality to emerge freely from the participants’ narratives. To ensure technical precision and scientific rigor, the script was reviewed by two herpetology specialists from UFOPA. The interviews took place at the residents’ homes with an average duration of 40 minutes; the answers were recorded in the questionnaire.

**Table 1:** Study instrument structure categorized by thematic axes.

Thematic Axis	Description of Variables and Questions	Objective
**Sociodemographic Profile**	Gender, age, education level, place of birth, length of residence, occupation (Q1), and religion (Q2).	To characterize the study population and identify social determinants of health.
**Epidemiological Experience and Ethnotaxonomy**	History of snakebite accidents (personal or family) and local vernacular nomenclature of the causative species (Q3).	To survey the incidence of accidents and the local knowledge regarding snake identification.
**Therapeutic Itineraries and Management**	Immediate post-bite procedures (Q4), perceived clinical sequelae (Q5)	To identify immediate actions and the use of traditional medicine, and perceived sequelae outcomes.
**Prevention and Information**	Measures adopted to avoid accidents (Q6) and primary sources of information and knowledge transmission (Q7).	To understand preventive practices and the flow of information within the community.

To achieve the widest possible sample coverage and ensure standardization in the application of the instrument, the interviews were conducted by a team of four previously trained researchers. Before the start of collection, a pre-test was conducted with two participants from different communities (not included in the final sample) to validate the clarity of the questions and ensure that the terms used were comprehensible and appropriate to the research objectives.

Audio recording was not used due to the refusal of the participants; this preference was ethically respected to ensure the spontaneity of the reports. Consequently, to ensure the reliable recording of data, the interviews were conducted only once by pairs of researchers (interviewer and note-taker) who took detailed and simultaneous field notes.

### Data analysis

Data analysis followed a mixed-methods approach. Initially, the narratives obtained from open-ended questions regarding epidemiological experiences, ethnotaxonomy, therapeutic itineraries, prevention methods, and forms of knowledge transmission were subjected to Content Analysis [33]. This process was carried out by a pair of researchers [BCFC, APSJ] to ensure inter-coder reliability. Any discrepancies in coding or categorization between the two researchers were resolved through technical consensus. The analysis was organized into three systematic stages: (1) pre-analysis, involving the organization and comprehensive reading of field notes; (2) material exploration, where narratives were inductively and deductively coded and categorized into the thematic axes described in Table 1; and (3) treatment and interpretation of results.

Once these categories were established, they were integrated with sociodemographic variables and processed using descriptive statistics in Microsoft Excel to calculate absolute and relative frequencies. For the final synthesis of ethnobiological knowledge, the “union of diverse individual competencies” method [34] was applied. This framework treats each participant’s report as a complementary part of a broader cultural system, consolidating a single inventory that represents the collective traditional knowledge of the Salvação community.

## Results

### Participant sociodemographic profile

A total of 39 residents participated in the study and have resided in the community for more than 10 years. Of these, 69% (n = 27) were men and 31% (n = 12) were women. Most participants, 84.6% (n = 33), were under 65 years old, and the highest education level reached was incomplete primary education, accounting for 35.9% (n = 14). Catholicism was the predominant religion at 51.2% (n = 20). Fishing represented the main subsistence activity for 51.2% (n = 20) of the participants, which is consistent with the broader subsistence structure in the community’s floodplain areas.

### Epidemiological experience and ethnotaxonomy

The majority of interviewees, 84.6% (n = 33), stated that they had already suffered or witnessed a family member or acquaintance in the community being affected by a snakebite. One riverine resident even mentioned that the entire family had been bitten by a snake: “Everyone at home has already been bitten by a snake: mom, dad, and my brother” (Interviewee 11). The elucidation of the species cited as the causes of envenomation in the community was based strictly on local knowledge and the perception of the participants, without the aid of projective tests or visual stimuli (such as photographs).

**Fig 2:**
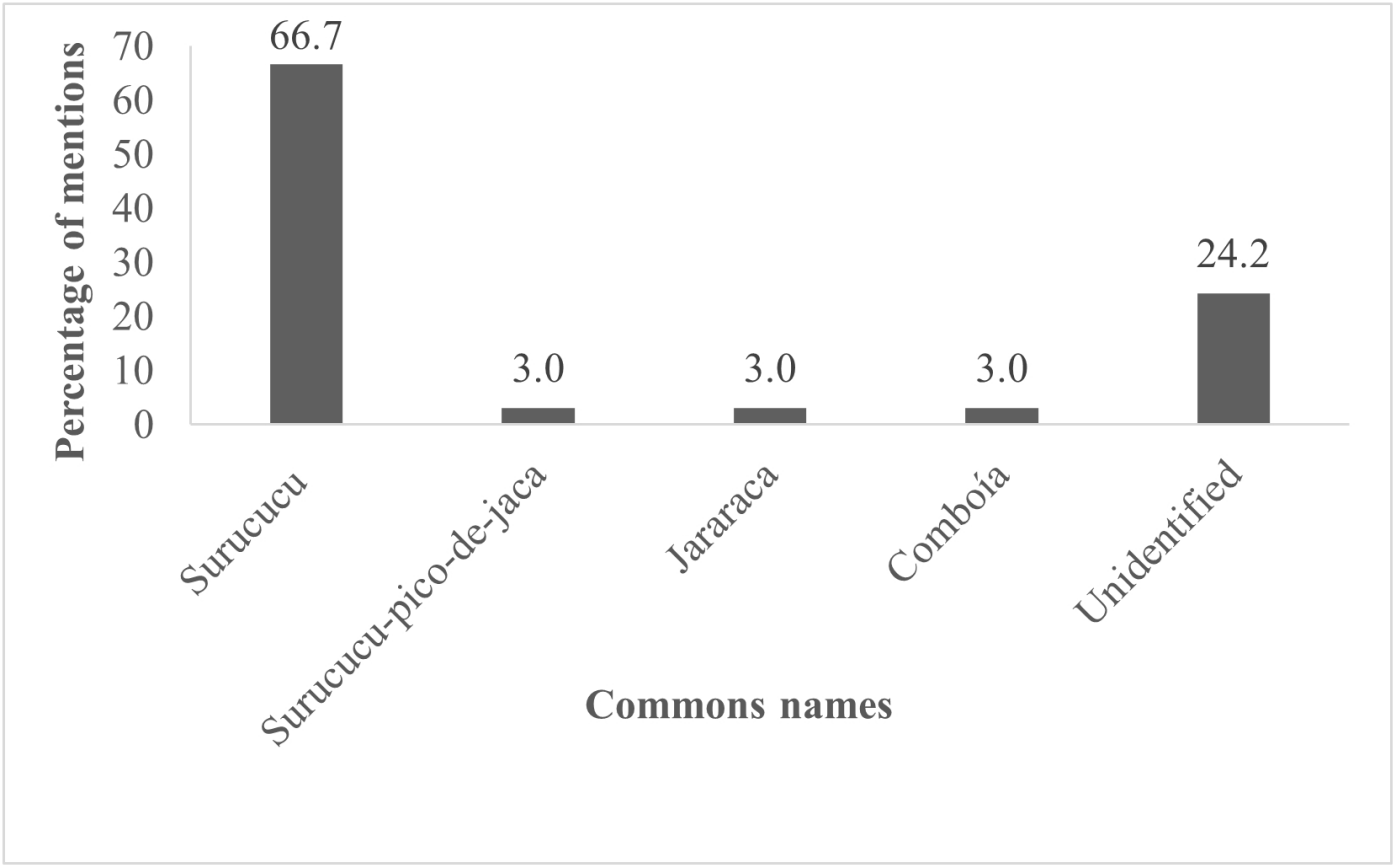
Ethnotaxonomy of snakes cited as most frequently involved in snakebite accidents in the community. Bar chart showing the popular snake names mentioned by riverine residents of the Salvação floodplain community (Alenquer, Pará, Lower Amazon, Brazil) as the species most associated with causing envenomation in the community. Values represent the percentage of interviewees who cited each name.

Popular names were recorded from a question asked during the interview about which snakes could have caused these accidents; based on this survey, the profile of the species that, in the community’s perception, are the main causes of these envenomations was established. The riverine residents demonstrated confidence in identification, with direct statements such as “It was the *surucucu*,” (Interviewee 31) leaving no room for doubt at the time of the report. On the other hand, almost a quarter of the participants, 24.2% (n = 8), were unable to identify the species involved. Other cited vernacular names included “*surucucu-pico-de-jaca*,” “*jararaca*,” and “*comboia*” (Fig 2).

### Therapeutic Itineraries and management

Hospital care was the most frequently reported treatment approach, at 33.3% (n = 13). This response was often accompanied by statements such as “They took them to Alenquer” (Interviewee 37), indicating that the victim was transported to the nearest medical center in the municipality of Alenquer (Pará), located approximately 50 minutes from the community. Other reports, such as “He went to the hospital to get the antivenom” (Interviewee 17), demonstrated the residents’ knowledge regarding the need for antivenom. Alongside conventional medical care, participants described the continued use of traditional therapies.

The most common traditional therapy was the application of “contraveneno” (anti-venom), described as “A medicine made with a mixture of plants” (Interviewee 15). The use of dolphin fat (*Inia geoffrensis* and/or *Sotalia fluviatilis*) was also central, being applied immediately after the bite, “He took the dolphin fat right away” (Interviewee 06) or during recovery. Less frequently reported practices included the ingestion of kerosene, “Before going to the hospital, he drank kerosene” (Interviewee 14), washing the wound with water, and applying parts of the snake’s body to the lesion site, “Take the snake’s brains and put them on top of the bite” (Interviewee 09) (Table 2). These practices reflect long-standing zootherapeutic traditions in the region.

**Table 2:** Treatment measures reported for snakebite incidents.

Treatment Measures	Participants (n)	Percentage (%)
Hospital care	13	33.3%
Traditional antidote	7	17.9%
Dolphin fat	5	12.8%
Kerosene ingestion	2	5.1%
Washing the wound with water	2	5.1%
Snake parts placed on the wound	1	2.6%
Did not answer	9	23.1%
**Total research participants**	**39**	**100%**

Traditional and biomedical treatment practices cited by residents of the Salvação floodplain community (Alenquer, Pará, Lower Amazon, Brazil) in response to snakebite events. Values represent the number and percentage of participants reporting each measure.

Persistent pain at the bite site was the most frequently reported long-term effect, at 25.6% (n = 10). Several participants associated the variation in the intensity of this pain with lunar phases, describing perceptions such as “I feel a sting at the bite site because of the moon” (Interviewee 21) or “It hurt when the moon time came” (Interviewee 16). Such reports reflect local ecological interpretations of natural cycles and their influence on healing processes. Other conditions perceived by the interviewees as sequelae of the envenomation included impaired vision (7.7%), lower limb atrophy (5.1%), and leg weakness (5.1%). Outcomes rarely attributed to the accident involved reports of softening of nails and teeth (2.6%), stroke (2.6%), and one fatal case (2.6%) (Table 3).

**Table 3:** Reported sequelae following snakebite incidents.

Sequelae of Snake Envenomation	Participants (n)	Percentage (%)
Pain at the envenomation site	10	25.6%
Impaired vision	3	7.7%
Atrophy of lower limbs (legs and foot)	2	5.1%
Weakened legs	2	5.1%
Softening of nails and teeth	1	2.6%
Stroke (Cerebrovascular Accident - CVA)	1	2.6%
Death	1	2.6%
There were no sequels	9	23.1%
Did not answer	10	25.6%
**Total research participants**	**39**	**100%**

Long-term health effects described by residents of the Salvação floodplain community (Alenquer, Pará, Lower Amazon, Brazil) after snakebite events. Values represent the number and percentage of participants reporting each sequela.

### Prevention and knowledge transmission

Most participants 59% (n = 23), reported adopting preventive measures: “I use boots, galoshes, gloves, and long pants” (Interviewee 27) or “Using boots and flashlights when leaving the house” (Interviewee 16). Some interviewees emphasized cautious movement in forested areas, 10.3% (n = 4): “You have to look and not touch; sometimes the bite happens without us seeing the snake” (Interviewee 08) or “Staying alert while walking along the paths” (Interviewee 10), while others highlighted the clearing of vegetation around residences, 7.7% (n = 3): “It is necessary to clear the land so snakes don’t appear” (Interviewee 13). However, 23.1% (n = 9) were unable to name any protective measure, suggesting uneven knowledge regarding prevention strategies.

**Fig 3:**
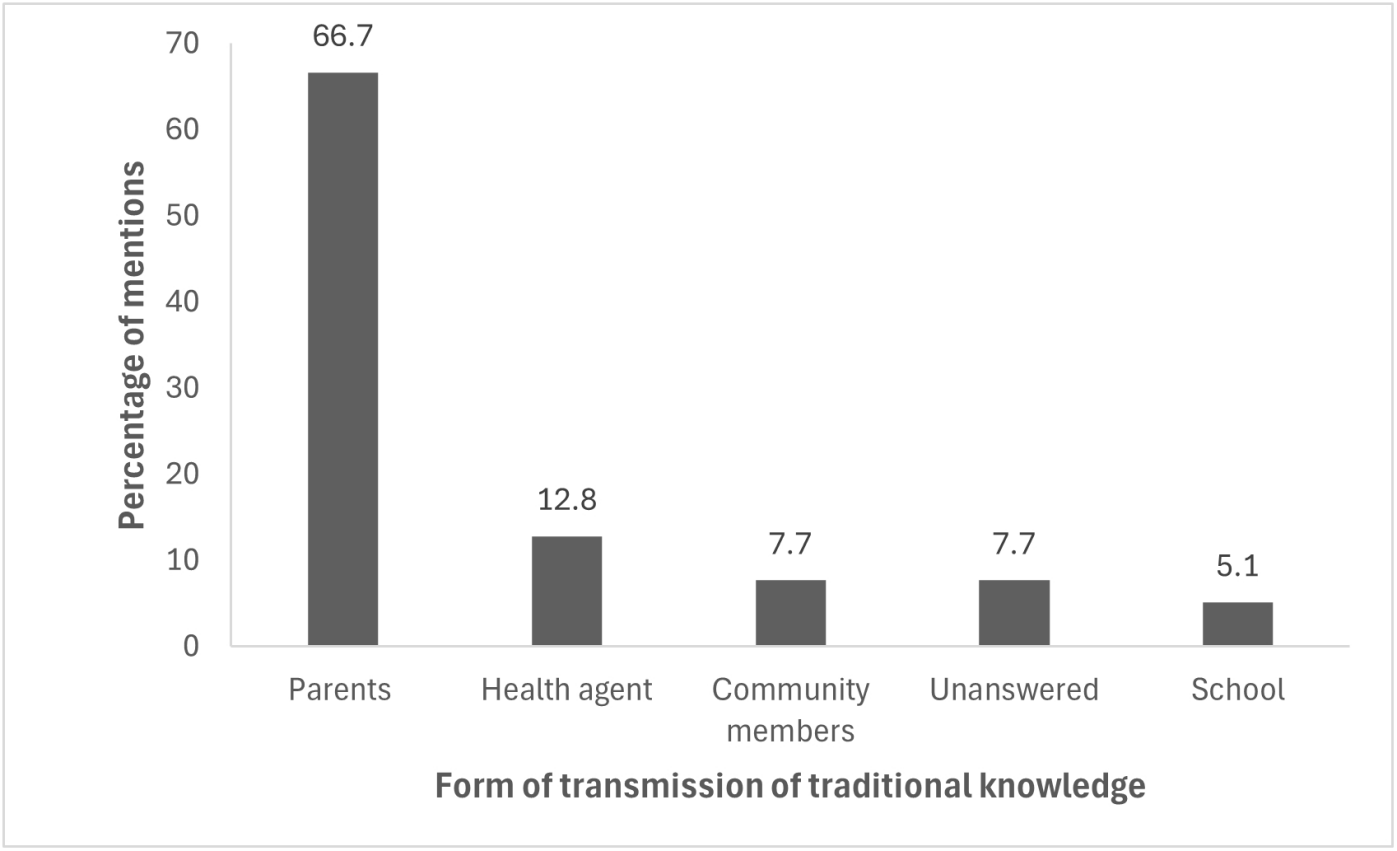
Sources of traditional knowledge transmission about snakebites. Proportion of community members in the Salvação floodplain community (Alenquer, Pará, Lower Amazon, Brazil) who reported different sources of learning regarding snakebite identification, treatment, and prevention.

Knowledge about snakebite accidents was reported as being transmitted primarily through family networks; 66.7% (n = 26) identified parents as their primary source of information. Health agents 12.8% (n = 5), schools 5.1% (n = 2), and other community members contributed on a smaller scale. A small proportion 7.7% (n = 3) did not identify any source of knowledge transmission (Fig 3). These findings point to the centrality of oral tradition in maintaining local health-related knowledge.

## Discussion

### Social determinants of health and local realities in the Amazon

The findings of this study suggest that the management of snakebite accidents in the Salvação community is a dynamic process, where cultural knowledge and the rhythms of the Amazonian floodplain appear to be as influential on clinical outcomes as access to antivenom. More than a simple cultural preference, the coexistence between formal and traditional medicine systems may reflect an ecological and social adaptation. Our data indicate that care-seeking behavior is not linear, but shaped by perceptions that at times challenge formal medical logic, highlighting the importance of integrating between local knowledge with public health strategies within this specific context.

The sociodemographic profile with low levels of formal education, observed in the Salvação community and in other regions of the Amazon [35], may be seen as a factor potentially contributing to the use of traditional treatments and a possible delay in seeking antivenom therapy. This appears to align with findings in Nepal [36], which suggest that textbooks often contain incorrect information, thus missing an educational opportunity to disseminate accurate information about prevention and conservation. Such observations encourage a reflection on World Health Organization (WHO) recommendations, emphasizing that health education strategies should be adapted to formal education at schools and universities [37] as well as local sociocultural realities [3].

### Ethnotaxonomy of snakes named as causing snakebite accidents

Local naming systems indicated significant discrepancies between vernacular and scientific classifications. In this context, “*surucucu*” and “*surucucu-pico-de-jaca*” generally refer to *Lachesis muta*, a species not found in the study area (APSJ pers obs). These terms were likely used to describe *Bothrops atrox*, the primary cause of envenomation in the Amazon [15]. Similar incongruities have been described in other Amazonian communities and suggest that local naming is often shaped by visual characteristics, perceived dangerousness, and collective memory, rather than taxonomic precision. Such divergences can complicate clinical assessment, highlighting the importance of culturally sensitive communication strategies during triage and treatment [38,39].

The difficulty in identifying species based on vernacular nomenclature observed in the Salvação community reflects a global challenge in the management of snakebite accidents. Just as the term “*surucucu*” is frequently used in the Amazon to describe *B. atrox*, ethnoherpetological studies in Nepal suggest that residents and even students use popular names that often do not correspond to scientific taxonomy, potentially hindering the recognition of venomous species. Risk perception in these contexts appears to be more closely linked to collective memory and visual characteristics than to biological precision [40]. This prevalence universality of functional naming systems reinforces the importance of health education strategies that integrate local names in a way that facilitates clinical triage.

### Therapeutic Itineraries: Integrating traditional and formal care

The predominance of hospital-seeking behavior contrasts with findings from more isolated communities in the Amazon, where geographical distance and transport limitations frequently delay or prevent access to conventional medical care [25]. The proximity of the Salvação community to the hospital in the municipality of Alenquer (Pará) may contribute to early treatment, an essential condition for reducing morbidity. However, traditional remedies continue to play a relevant role in local therapeutic repertoires. The use of dolphin fat (*Inia geoffrensis* and/or *Sotalia fluviatilis*) reproduces patterns described elsewhere in the region [41] and illustrates how traditional zootherapeutic knowledge persists, despite conservation concerns identified by the IUCN [42].

The persistence of traditional practices, such as the use of dolphin fat in the Amazon, aligns with realities observed in Ghana and Nepal. In Ghana, traditional healers play a central role as primary care providers, using remedies that often precede going to the hospital [43]. Similarly, in Nepal, reliance on traditional healers is fueled by limited accessibility to conventional medical care and its high costs [19]. These global practices may be seen not only as cultural choices, but as adaptation mechanisms in areas where the formal health system is perceived as distant. Recognizing these actors could be a key strategy for creating bridges between traditional medicine and the formal medical system, potentially reducing the time to reach the hospital.

The mention of kerosene and snake-derived materials as treatments is similar to ethnographic observations from other Amazonian and indigenous contexts [44,45]. These practices suggest the adaptive nature of traditional medicine within these populations. However, the use of toxic or harmful substances highlights informational gaps that may worsen clinical outcomes. Health education interventions that incorporate local explanatory models, while providing clear guidance on harmful practices, could be essential strategies for this region [35].

These gaps in knowledge regarding first aid are not isolated issues of Amazonian communities and align with practices identified in Malawi and Ghana, such as the use of drinks based on various herbs (some mixed with alcoholic beverages), the use of incisions, suction, black stones, and tourniquets. This indicates that the lack of standardized information among community members and potentially among local health providers can delay the search for formal medical care and can lead to medical complications due to the use of non-sterile materials in cases of incision, as well as systemic damage from the use of alcohol and toxic substances [43,46].

### Ethnobiological perspectives on sequelae and environmental rhythms

Persistent pain was the most frequently perceived sequela and was associated with changes in lunar cycles. Similar results were observed in a study in the Southeast region of Brazil, in the Bom Jardim community, where the influence of lunar cycles on the outcomes of family farming practices was documented. These interpretations suggest that the perceived power of lunar cycles presents itself under different perspectives, shaping local understanding of biological changes in the body, and at others influencing environmental practices such as agriculture [47]. The complex relationship between the riverine population and the ecosystem points to a profound ethnobiological knowledge regarding the perception of snakebite accidents, which intrinsically connects snake-man-environment.

From this perspective, the perception of sequelae appears not as an isolated event in the body, but a phenomenon tuned to the rhythms of nature. The influence of sociocultural and ecological factors on the experience of envenomation finds global parallels, such as investigations conducted in Nepal, where geographical determinants and local perceptions are key to understanding how communities interpret and react to snakebite accidents [19]. Other reports, including vision problems, limb weakness, and perceptions of conditions similar to stroke, although less frequent, are perceived by the community as part of the spectrum of possible complications.

### Barriers to prevention and the centrality of oral tradition

Although the use of boots and flashlights was widely recognized as a preventive measure by the riverine residents of the Salvação community, a significant gap is noted observed between theoretical knowledge and daily practice. Reports of accidents in backyards and inside homes, which often intensify during the flood period when snakes seek refuge, indicate that risk can be present in various locations and extends beyond the formal work environment. This ‘fine line’ between knowing and doing reflects a global challenge in snakebite accident prevention.

Studies in Southeast Africa and South Asia reinforce that even with an understanding of risks, factors such as temperature, equipment cost, and the nature of domestic activities can limit continuous adherence to safety items [36,46]. Thus, the data suggest that prevention should not focus solely on the distribution of equipment but could also involve the adaptation of dwellings and on educational strategies that consider local ecology and the habits of riverine life, where danger may reside within the very domesticity of the space.

Oral transmission, especially through parents, was the primary way of transferring knowledge. Oral narratives constitute both memory and practice and represent a significant channel for the dissemination of preventive behaviors and treatment guidelines. This centrality of oral tradition finds parallels in communities in West Africa and South Asia, where trust in family networks and local leaders can at times be as influential as formal public health campaigns.

This reinforces the perspective that successful interventions are often those that utilize trusted messengers and local leaders to translate formal medical knowledge into local narratives [21,36]. Public health initiatives that collaborate with community leaders, elders, and family networks may, therefore, increase the efficacy and acceptability of snakebite prevention and early care strategies [48,49].

Although the findings of this study are anchored in the specific reality of the Salvação community, the observed patterns, such as the influence of water seasonality on domestic risk and the persistence of traditional healing systems, may reflect dynamics common to other floodplain communities in the Lower Amazon. However, given the vast cultural and geographical diversity of the Amazon region, the generalization of these data to upland (terra firme) contexts or other regions should be approached with caution. This study serves as a case study for understanding the complexity of human-snake interactions in similar riverine environments.

### Limitations

Despite the contributions of this study to the understanding of snakebite accidents in the Lower Amazon floodplain, some limitations must be considered. First, the sample size was influenced by logistical and geographical challenges inherent to working in remote communities, where field permanence was limited by the availability of local shelter. Furthermore, the reserved profile of part of the population required rigorous ethical care, priority was given to establishing prior dialogue and trust before interviews. While this approach reduced the total number of participants, it ensured the authenticity and depth of the reports.

Regarding data accuracy, although transcripts were not returned to participants due to logistical constraints, information was validated through real-time oral confirmation during the dialogues. To mitigate potential observer bias and ensure accuracy in the absence of audio recordings, field notes were meticulously recorded immediately after each interaction. Whenever ambiguity arose, researchers immediately sought further clarification from the participant to ensure that the records faithfully represented their narratives.

This real-time validation (member checking) allowed for the immediate correction of any misinterpretations. Finally, concerning snake identification, the data were based on the participants’ perception and vernacular nomenclature (ethnotaxonomy) without taxonomic confirmation through collected specimens. Additionally, recall bias must be considered, as reports on symptoms and time to treatment depend on the participants’ recollection of past events.

## Conclusion

This study indicates that the management of snakebite accidents in the Amazonian floodplain is guided by a complex ethnobiological knowledge, where the perception of the disease and its treatment is integrated into a dynamic relationship between humans, snakes, and the environment. The persistence of traditional practices and the association of sequelae with natural cycles should not be seen as a lack of knowledge, but as models of care that provide cultural safety to families in regions where the formal medical system is still perceived as distant.

## Supporting information

S1 Checklist COREQ Checklist. (PDF)

S2 Checklist Inclusivity in global research. (PDF)

S1 Data Spreadsheet containing the analysis of the interview data. (XLSX)

S1 Text Interview guide used for data collection. (PDF)
