# Supplementary material for: Snakebite envenomation and community responses in an Amazonian floodplain: Public health and ethnobiological perspectives
Source: PLOS Glob Public Health. 2026 Apr 15;6(4):e0006310. doi: 10.1371/journal.pgph.0006310 (PMC13082696; doi:10.1371/journal.pgph.0006310)
Supplement: S1 Checklist — (PDF) [file pgph.0006310.s001.pdf]

S1 Checklist. COREQ (Consolidated Criteria for Reporting Qualitative Research) Checklist. The lines refer to the clean manuscript.		
**Item No.**	**Item Description**	**Location in manuscript**
**Domain 1: Research team and reflexivity**		
1	Interviewer/facilitator	138,139
2	Credentials	139,140
3	Occupation	139,140
4	Gender	139
5	Experience and training	141
6	Relationship established	142-144
7	Participant knowledge of the interviewer	145-148
8	Interviewer characteristics	148-151
**Domain 2: Study design**		
9	Methodological orientation	130-136
10	Sampling	185
11	Method of approach	203
12	Sample size	196
13	Non-participation (refusals)	197
14	Setting of data collection	214
15	Presence of non-participants	203
16	Description of sample	256,257
17	Interview guide	219-221
18	Repeat interviews	226
19	Audio/visual recording	223,224
20	Field notes	227
21	Duration	215
22	Data saturation	196
23	Transcripts returned	473-478
**Domain 3: Analysis and findings**		

24	Data coders	233
25	Description of the coding tree	255,263,285,321
26	Derivation of themes	208-212
27	Software	239
28	Participant checking	477-478
29	Quotations presented	Direct quotations from participants are presented to illustrate key findings, identified by alphanumeric codes to preserve anonymity."Interviewee 01"
30	Data and findings consistent	342
31	Clarity of major themes	255,263,285,321
32	Clarity of minor themes	343,360,379,412,431
