# Supplementary material for: Snakebite envenomation and community responses in an Amazonian floodplain: Public health and ethnobiological perspectives
Source: PLOS Glob Public Health. 2026 Apr 15;6(4):e0006310. doi: 10.1371/journal.pgph.0006310 (PMC13082696; doi:10.1371/journal.pgph.0006310)
Supplement: S2 Checklist — (PDF) [file pgph.0006310.s002.pdf]

Inclusivity in global research

PLOS' policy on inclusivity in global research aims to improve transparency in the reporting of research performed outside of researchers' own country or community and ensures that PLOS publications reporting global research adhere to high standards for research ethics and authorship. Authors of relevant research articles may be asked to complete the questionnaire below, which outlines ethical, cultural, and scientific considerations specific to inclusivity in global research. This questionnaire may be requested when researchers have travelled to a different country to conduct research, if research uses samples collected in another country, research with Indigenous populations or their lands, or if research is on cultural artefacts. Researchers travelling to another country solely to use laboratory equipment will not normally be required to complete the questionnaire. However, the questionnaire can be requested at the journal's discretion for any submission – if you have been requested to complete this questionnaire by the PLOS journal you submitted to, please do so.

Please complete the questionnaire below and include this as a Supporting Information file with your manuscript. Note that if your paper is accepted for publication, this checklist will be published with your article in the supporting information files. Please ensure that you reference the checklist in the main body of your manuscript. We suggest adding a subsection 'Inclusivity in global research' to your Methods section and adding the following sentence: "Additional information regarding the ethical, cultural, and scientific considerations specific to inclusivity in global research is included in the Supporting Information (SX Checklist)"

The questions have been designed to be applicable to a wide range of study types, and there are subsections for both human subjects research and non-human subjects research. If any of the questions are not relevant to your research please mark them as "N/A" as appropriate.

Ethical considerations, permits and authorship

This section is applicable to all research types.

Provide details as to who granted permissions and/or consent for the study to take place in the Methods section of your manuscript. This should include the names of **all** ethics boards, governmental organizations, community leaders or other bodies that provided approval for the study. If individuals provided approval refer to these people by their role or title but do not list their name(s).

Reported on page number: Page 10 and 11

If there were any deviations from the study protocol after approval was obtained please provide details of these changes in the Methods section of your manuscript.

Reported on page number: Page 9 and 10

Did this study involve local collaborators that are residents of the country where the research was conducted

or members of the community studied? If you do not have any authors from said communities, please provide an explanation for this below.

The research team is entirely composed of researchers from the Universidade Federal do Oeste do Pará (UFOPA), which is the local institution responsible for the region where the study was conducted. While residents of the Salvação community were not directly involved as co-authors in the manuscript's writing, they acted as essential collaborators and key informants. Their involvement was prioritized during data collection and through constant dialogue with community leaders, ensuring that traditional knowledge was respected and accurately documented. The decision not to include community members as authors is based on the fact that the intellectual design and academic writing were led by the university team, but the community's contribution is explicitly recognized in the Acknowledgments. Furthermore, this study is part of a broader doctoral project that includes a planned communication of results to the community, ensuring that the findings are shared with the participants in an accessible and culturally appropriate format.

Everyone listed as an author should meet PLOS' criteria for authorship and all individuals who meet these criteria should be included in the author byline, rather than the acknowledgements. For further information please see the journal's Authorship Policy.

Human subjects research (e.g. health research, medical research, cross-cultural psychology)

Did you obtain written informed consent from a representative of the local community or region before the research took place? How did you establish who speaks for the community? Details of written informed consent obtained from study participants should be reported separately in the Methods section of your manuscript.

Yes, we obtained the free and informed consent in writing from the community leader, as well as from all those interviewed from the Salvation Community. On our first visit to the community, we immediately sought to find out who the community leader was and requested a meeting with him. The details of the Free and Informed Consent Form can be found on page 11.

How did members of the local community provide input on the aims of the research investigation, its methodology, and its anticipated outcome(s)?

The members of the Salvation Community contributed by making themselves available to report their perceptions about snakebite accidents in the community through our semi-structured interview methodology. The results allowed us to construct, from the perceptions heard, the community's view regarding snakebite accidents.

When engaging with the local community, how did you ensure that the informed consent documents and other materials could be understood by local stakeholders?

The document was structured as objectively as possible, avoiding overly technical terms so as not to hinder the understanding of the interviewees. Before each interview, we read the document aloud and then summarized it in our own words, emphasizing what had been read.

Will the findings of the research be made available in an understandable format to stakeholders in the community where the study was conducted (e.g. via a presentation, summary report, copies of publications, etc.)? Please provide details of how this will be achieved.

The results will be sent in banner format containing illustrative images of snakes that cause accidents in the region. To make these images more educational, they will also be provided in paper calendar format (commonly used by community members to check dates throughout the year). We devised this strategy so that community members can become more familiar with the images.

Non-human subjects research using specimens/ animals collected as part of the study, or those housed in archival collections. Examples include archaeology, paleontology, botany and zoology.

Did the permission you obtained from a local authority to perform the study include an agreement on access to outputs and benefit sharing? This may include procedures to enable fair distribution of the benefits and resources arising from the research performed. Please include any details of Prior Informed Consent and Benefit Sharing Agreements obtained. These may be required by field-specific regulations, for example the Convention on Biological Diversity (CBD) and the associated Nagoya Protocol.

N/A

If the material used in your study was imported, please A) provide the year it was imported and B) indicate whether permits were obtained to import/export the materials used, C) provide details of any permits obtained. If this information is not available, please indicate this.

N/A

If you used archival specimens, please state how the material used in your study was acquired by the institute it is held in and provide details of any permits obtained for the original excavations/ sample collection. If this information is not available, please indicate this.

N/A

How was the potential cultural significance of the materials collected in your study to local communities considered in your research design? Were Indigenous peoples and/or local researchers and institutions involved with archaeological excavations / collection of specimens? If so, please provide a description of their involvement.

N/A

If your manuscript includes photographs of human remains please indicate whether authors obtained permission from descendants or affiliated cultural communities to do so.

N/A
