# Supplementary material for: Snakebite envenomation and community responses in an Amazonian floodplain: Public health and ethnobiological perspectives
Source: PLOS Glob Public Health. 2026 Apr 15;6(4):e0006310. doi: 10.1371/journal.pgph.0006310 (PMC13082696; doi:10.1371/journal.pgph.0006310)
Supplement: S1 Text — (PDF) [file pgph.0006310.s004.pdf]

UNIVERSIDADE FEDERAL DO OESTE DO PARÁ

ROTEIRO ENTREVISTAS

Entrevistador: ________________________________ Data:_____________________

Comunidade: ____

Categoria 1- Perfil Sociodemográfico

Objetivo: Caracterizar a população estudada e identificar os determinantes sociais.

Sexo: F ( ) M ( )

Idade:____________________

Grau de escolaridade: _________________________________

Onde você nasceu? _________________________________

Quanto tempo mora na comunidade? _____________________________

1-Qual função você ocupa na comunidade?

____

2- Qual a sua religião?

____

Categoria 2: Experiência Epidemiológica e Etnotaxonomia

Objetivo: Levantar o histórico de acidentes e a nomeação de espécies locais que na percepção dos ribeirinhos são as serpentes causadoras desses acidentes.

3- Você ou alguém da sua família já foi picado por uma cobra? Qual o nome da cobra que pode ter causado esses acidentes?

____

____

____

____

____

Categoria 3: Itinerários terapêuticos e manejo dos acidentes

Objetivos: Identificar ações imediatas, o uso da medicina tradicional e as sequelas percebidas.

4- Qual o procedimento adotado logo após a picada?

____

____

____

____

____

5- Após o acidente por picada de cobra, a pessoa em questão, teve sequelas? Quais?

____
____
____
____
____

Categoria 4: Prevenção e Informação

Objetivo: Compreender os comportamentos preventivos e como o conhecimento é transmitido dentro da comunidade.

6-Você sabe dizer quais as medidas de prevenção para evitar acidentes com cobras?

____
____
____
____
____

7- Onde você obteve informações sobre o que fazer quando ocorre acidente com cobra?

( ) Agentes de saúde

( ) Com os pais

( ) Na escola

( ) Palestra

( ) Televisão

( ) Igreja

( ) Internet

( ) Outro:

____
____
____
____
____
____

Translation

S2 Questionnaire

Category 1: Sociodemographic Profile

Objective: To characterize the study population and identify social determinants.

Gender: F ( ) / M ( )

Age:______________

Education level: ________________

Place of birth: __________________

Length of residence in the Community: __________________

Q1: What is your role or occupation in the community?

Q2: What is your religion?

Category 2: Epidemiological Experience and Ethnotaxonomy

Objective: To survey the history of accidents and the local identification of snake species.

Q3: Have you or anyone in your family ever been bitten by a snake? What is the name of the snake that may have caused these accidents?

Category 3: Therapeutic Itineraries and Management

Objective: To identify immediate actions, the use of traditional medicine, and perceived clinical outcomes.

Q4: What procedure was adopted immediately after the bite?

Q5: After the snakebite accident, did the person in question have any sequelae (long-term effects)? Which ones?

Q6: Do you know of any home treatments or traditional remedies for snakebites? Which ones?

Category 4: Prevention and Information

Objective: To understand preventive behaviors and how knowledge is transmitted within the community.

Q7: Can you describe the preventive measures to avoid snakebite accidents?

Q8: Where did you obtain information about what to do when a snakebite accident occurs?
